# Which one is better for refractory/relapsed acute B-cell lymphoblastic leukemia: Single-target (CD19) or dual-target (tandem or sequential CD19/CD22) CAR T-cell therapy?

**DOI:** 10.1038/s41408-023-00819-5

**Published:** 2023-04-24

**Authors:** Sining Liu, Xinyue Zhang, Haiping Dai, Wei Cui, Jia Yin, Zheng Li, Xiao Yang, Chunxiu Yang, Shengli Xue, Huiying Qiu, Miao Miao, Suning Chen, Zhengming Jin, Chengcheng Fu, Caixia Li, Aining Sun, Yue Han, Ying Wang, Lei Yu, Depei Wu, Qingya Cui, Xiaowen Tang

**Affiliations:** 1 National Clinical Research Center for Hematologic Diseases, Jiangsu Institute of Hematology, The First Affiliated Hospital of Soochow University, Suzhou, 215006 China; 2 Institute of Blood and Marrow Transplantation, Collaborative Innovation Center of Hematology, Soochow University, Suzhou, 215123 China; 3 Shanghai Unicar-Therapy Bio-medicine Technology Co., Ltd, Shanghai, 201203 China

**Keywords:** Immunotherapy, Acute lymphocytic leukaemia, Bone marrow transplantation

## Abstract

CD19 chimeric antigen receptor (CAR) T-cell therapy has shown great success against B-cell acute lymphoblastic leukemia (B-ALL). Tandem and sequential CD19/CD22 dual-target CAR T-cell therapies have been developed to reduce the possibility of CD19-negative relapse; however, the superior strategy is still uncertain. This study screened 219 patients with relapsed/refractory B-ALL who were enrolled in clinical trials of either CD19 (NCT03919240) or CD19/CD22 CAR T-cell therapy (NCT03614858). The complete remission (CR) rates in the single CD19, tandem CD19/CD22, and sequential CD19/CD22 groups were 83.0% (122/147), 98.0% (50/51), and 95.2% (20/21), respectively (single CD19 vs. tandem CD19/CD22, *P* = 0.006). Patients with high-risk factors achieved a higher rate of CR in the tandem CD19/CD22 group than in the single CD19 group (100.0% vs. 82.4%, *P* = 0.017). Tandem CD19/CD22 CAR T-cell therapy was one of the significant favorable factors in the multivariate analysis of the CR rate. The incidence of adverse events was similar among the three groups. Multivariable analysis in CR patients showed that a low frequency of relapse, a low tumor burden, minimal residual disease-negative CR and bridging to transplantation were independently associated with better leukemia-free survival. Our findings suggested that tandem CD19/CD22 CAR T-cell therapy obtains a better response than CD19 CAR T-cell therapy and a similar response to sequential CD19/CD22 CAR T-cell therapy.

## Introduction

Relapsed/refractory (R/R) B-cell acute lymphoblastic leukemia (B-ALL) is associated with a poor response to salvage therapies and a dismal prognosis [1–5]. Chimeric antigen receptor (CAR) T-cell therapy has achieved great advances in recent years. CD19 is a nearly ideal target antigen for B-ALL because of its homogeneous expression [6]. Multiple clinical trial results have shown a 68%–93% remission rate after CD19 CAR T-cell therapy in children and adults with R/R B-ALL [7–12]. However, it has not been able to maintain durable remission in most patients. A long-term follow-up study reported a median event-free survival (EFS) time of 6.1 months [11]. CD19-negative relapse is one of the major causes of therapeutic failure, occurring in 25%–42% of responding patients [10, 11, 13].

Dual-target CAR T-cells have been developed to reduce the possibility of CD19-negative relapse. CD22 is another member of the B-cell antigen family whose tissue distribution is similar to that of CD19 [14, 15]. Dual-target CAR T-cells can be applied using several combination strategies, including cocktail CD19/CD22 CAR T-cell therapy, sequential infusion and bispecific CAR T-cell products [13, 16–21]. The optimal combination strategy for CAR T-cells with different target antigens is still uncertain.

Therefore, we compared the efficacy and safety of single CD19, tandem CD19/CD22 and sequential CD19/CD22 CAR T-cell therapies in R/R B-ALL at our institution.

## Methods

### Patients

**Fig. 1:**
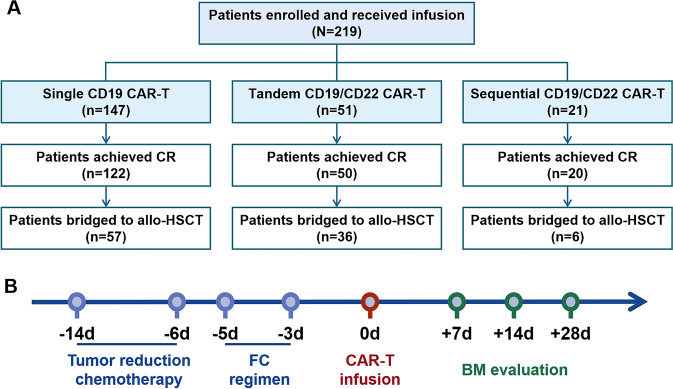
Retrospective study cohort. **A** A total of 219 patients with R/R B-ALL successfully received CAR T-cell treatments (147 patients received single CD19 CAR T-cell therapy, 51 patients received tandem CD19/CD22 CAR T-cell therapy, and 21 patients received sequential CD19/CD22 CAR T-cell therapy) were screened in this study. Ninety-nine patients who achieved remission with CAR T-cell therapy bridged to allo-HSCT. **B** Schematic of CAR T-cell therapy regimen.

Between August 2018 and October 2021, a total of 219 patients with R/R B-ALL who successfully received CAR T-cell treatments were screened in this study (Fig. 1A). Among them, 147 patients received single CD19 CAR T-cell therapy, 51 patients received tandem CD19/CD22 CAR T-cell therapy, and 21 patients received sequential CD19/CD22 CAR T-cell therapy. All patients were enrolled in CD19 CAR T-cell clinical trials (NCT03919240) or CD19/CD22 CAR T-cell clinical trials (NCT03614858). This study was approved by the ethics committee of The First Affiliated Hospital of Soochow University, and all patients provided written informed consent.

### Treatment protocol

After chemotherapy to reduce the tumor burden, all patients received lymphodepletion with fludarabine (30 mg/m^2^/d) and cyclophosphamide (300 mg/m^2^/d) based conditioning regimens on day −5 to −3. CAR T-cells were then infused at day 0 (Fig. 1B). The median dose of CAR T-cells was 5 (5–20) × 10^6^ cells/kg in the single CD19 group, 10 (5–20) × 10^6^ cells/kg in the tandem CD19/CD22 group, and 10 × 10^6^ cells/kg in the sequential CD19/CD22 group.

### Definitions

Patients with high-risk factors were defined in accordance with the National Comprehensive Cancer Network guidelines, version 3.2020. Complete remission (CR) was defined as less than 5% blasts in bone marrow morphology and no extramedullary disease (EMD). Minimal residual disease (MRD)-negative status was defined as a leukemic cell count below the sensitivity threshold of 1 × 10^−^^4^ (0.01%) per bone marrow mononuclear cell (MNC) by multiparameter flow cytometry [22, 23]. Cytokine release syndrome (CRS) was defined as “a disorder characterized by fever, tachypnea, headache, tachycardia, hypotension, rash, and/or hypoxia caused by the release of cytokines” [24]. Immune effector cell-associated neurotoxicity syndrome (ICANS) was defined as “a disorder characterized by a pathologic process involving the central nervous system following any immune therapy that results in the activation or engagement of endogenous or infused T cells and/or other immune effector cells. Symptoms or signs can be progressive and may include aphasia, altered level of consciousness, impairment of cognitive skills, motor weakness, seizures, and cerebral edema” [24]. CRS and ICANS were assessed according to the American Society for Transplantation and Cellular Therapy Consensus Grading [24]. Hemophagocytic histiocytosis (HLH) was diagnosed according to the HLH-2004 criteria [25]. The diagnosis of tumor lysis syndrome (TLS) was based on the Cairo-Bishop criteria [26]. Other organ toxicities were graded according to the Common Terminology Criteria for Adverse Events Version 5.0. Overall survival (OS) was the time from CAR T-cells infusion to death for any reason. Leukemia-free survival (LFS) was the duration from the day of CR after CAR T-cell therapy to leukemia relapse, death or the last follow-up. Patients alive in CR at the time of last follow-up were administratively censored.

### Statistical analysis

The differences among the three groups were analyzed by one-way ANOVA for continuous variables, and by Chi-square test or Fisher’s exact test for categorical variables. The Bonferroni method was used for pairwise comparisons. Multivariate logistic regression was applied to analyze CR prognostic factors. The probabilities of OS and LFS were calculated by the Kaplan-Meier method. The cumulative incidence of relapse (CIR) was estimated using a competing risk model, with non-relapse mortality as a competing risk factor. Multivariate analyses of prognostic factors for LFS were conducted with Cox regression. *P* values (2-tailed) <0.05 were considered statistically significant. All statistical analyses were performed with SPSS version 22, GraphPad Prism version 8.3.0 and R version 3.6.2.

## Results

### Patient characteristics

A total of 219 R/R B-ALL patients who received CAR T-cell treatments were enrolled in our retrospective study, comprising 147 patients in the single CD19 group, 51 patients in the tandem CD19/CD22 group and 21 patients in the sequential CD19/CD22 group. The baseline characteristics of the three groups are summarized in Supplementary Table 1.

There were 106 males and 113 females. The median age of the patients was 31 years (range 6–72 years). Fifty patients (22.8%) had primary refractory disease at the time of enrollment, 120 patients (54.8%) were experiencing the first relapse, and 49 patients (22.4%) were experiencing the second or more relapse. A total of 118 patients (53.9%) had previously received more than 4 therapies, and 46 patients (21.0%) had previously undergone allogeneic hematopoietic stem cell transplantation (allo-HSCT). Before lymphodepletion, 89 patients (40.6%) had a high tumor burden with over 20% blasts in bone marrow by morphology. Ninety patients (41.1%) had MRD with bone marrow blasts in the range of 0.01% to less than 5%. Twenty patients (9.1%) had EMD. Twenty-two patients (10.0%) harbored a complex karyotype. The *BCR/ABL1* fusion gene was detected in 64 patients (29.2%), 30 of whom were accompanied by the *T315I* mutation. Ten patients (4.6%) were classified as Ph-like ALL, and 12 patients (5.5%) harbored a *KMT2A* rearrangement. *TP53* mutation was detected in 17 patients (7.8%).

The proportion of patients with high-risk cytogenetic or genetic characteristics was similar in the three groups (50.3%, 54.9% and 28.6%, respectively; *P* = 0.116), defined as complex karyotype, *KMT2A* rearranged, *BCR-ABL1*, Ph-like ALL, mutated *TP53* or *IKZF1*. Among 64 patients with *BCR/ABL1*, 48 were in the single CD19 group, 16 were in the tandem CD19/CD22 group, and no patient was in the sequential CD19/CD22 group (*P* = 0.008). Otherwise, the baseline characteristics of the three groups were similar.

### Tandem CD19/CD22 CAR T-cell therapy produced a better therapeutic response than single CD19 CAR T-cell therapy

**Fig. 2:**
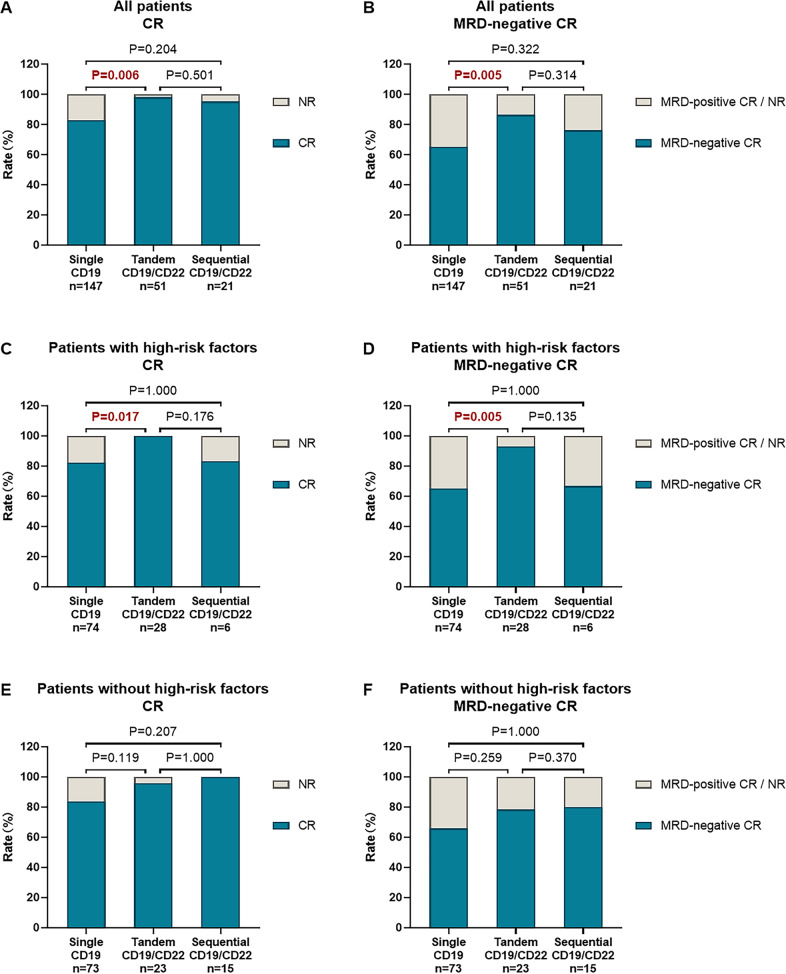
Response to CAR T-cell therapy. **A** CR and **B** MRD-negative CR rate in all patients. **C** CR and **D** MRD-negative CR rate in patients with high-risk cytogenetic and genetic characteristics. **E** CR and **F** MRD-negative CR rate in patients without high-risk cytogenetic and genetic characteristics.

The CR rate of patients in the tandem CD19/CD22 group was significantly higher than that in the single CD19 group (98.0% vs. 83.0%, *P* = 0.006), and was similar to that in the sequential CD19/CD22 group (98.0% vs. 95.2%, *P* = 0.501) (Fig. 2A). We further performed subgroup analyses to explore which patients benefited from dual CD19/CD22 CAR T-cell therapy (Table 1). Compared to the single CD19 group, the tandem CD19/CD22 group had an increased CR rate in patients with the following characteristics: no history of allo-HSCT (84.5% vs. 100.0%, *P* = 0.004), no EMD (88.0% vs. 100.0%, *P* = 0.013), a high tumor burden (78.2% vs. 100.0%, *P* = 0.014), and no complex karyotype (83.0% vs. 97.8%, *P* = 0.011). High-risk patients in the tandem CD19/CD22 group showed a higher CR rate than those in the single CD19 group (100.0% vs. 82.4%, *P* = 0.017) and a similar CR rate to those in the sequential CD19/CD22 group (100.0% vs. 83.3%, *P* = 0.176) (Fig. 2C). In patients without high-risk factors, there was no significant difference in the CR rate among the three groups (83.6%, 95.7% and 100.0%, respectively; *P* = 0.137) (Fig. 2E).

**Table 1:** Subgroup analysis of CR rate.

Characteristic	Single CD19 *n* = 147	Tandem CD19/CD22 *n* = 51	Sequential CD19/CD22 *n* = 21	*P* value
	*n*/*N* (%)	*n*/*N* (%)	*n*/*N* (%)	
Gender				
Male	54/68 (79.4%)	29/30 (96.7%)	7/8 (87.5%)	0.062
Female	68/79 (86.1%)	21/21 (100.0%)	13/13 (100.0%)	0.100
Age (y)				
≤14	10/11 (90.9%)	7/7 (100.0%)	2/2 (100.0%)	1.000
15–34	57/69 (82.6%)	25/26 (96.2%)	11/12 (91.7%)	0.183
≥35	55/67 (82.1%)	18/18 (100.0%)	7/7 (100.0%)	0.108
Disease status				
Refractory	30/32 (93.8%)	15/15 (100.0%)	3/3 (100.0%)	1.000
First relapse	67/80 (83.8%)	27/28 (96.4%)	12/12 (100.0%)	0.107
Second or more relapse	25/35 (71.4%)	8/8 (100.0%)	5/6 (83.3%)	0.266
Course of prior therapy				
≤3	62/72 (86.1%)	23/23 (100.0%)	6/6 (100.0%)	0.174
≥4	60/75 (80.0%)	27/28 (96.4%)	14/15 (93.3%)	0.068
Prior allo-HSCT				
Yes	24/31 (77.4%)	8/9 (88.9%)	6/6 (100.0%)	0.605
No	98/116 (84.5%)^#^	42/42 (100.0%)^#^	14/15 (93.3%)	**0.009**
EMD				
CNSL	2/5 (40.0%)	0/0	0/0	—
Other EMD	3/9 (33.3%)	5/6 (83.3%)	0/0	0.119
Negative	117/133 (88.0%)^#^	45/45 (100.0%)^#^	20/21 (95.2%)	**0.020**
BM blasts by morphology				
<5%	56/64 (87.5%)	19/19 (100.0%)	7/7 (100.0%)	0.341
5-20%	23/28 (82.1%)	6/7 (85.7%)	5/5 (100.0%)	0.813
≥20%	43/55 (78.2%)^#^	25/25 (100.0%)^#^	8/9 (88.9%)	**0.021**
Complex karyotype				
Yes	10/12 (83.3%)	6/6 (100.0%)	3/4 (75.0%)	0.556
No	112/135 (83.0%)^#^	44/45 (97.8%)^#^	17/17 (100.0%)	**0.006**
Fusion gene				
*BCR-ABL1*	40/48 (83.3%)	16/16 (100.0%)	0/0	0.185
Ph-like	4/4 (100.0%)	5/5 (100.0%)	1/1 (100.0%)	—
*KMT2A* rearrangement	8/9 (88.9%)	1/1 (100.0%)	2/2 (100.0%)	1.000
Other fusion genes	4/6 (66.7%)	4/4 (100.0%)	2/2 (100.0%)	0.636
Negative	66/80 (82.5%)	24/25 (96.0%)	15/16 (93.8%)	0.168
Gene mutation				
*T315I*	21/25 (84.0%)	5/5 (100.0%)	0/0	1.000
*TP53*	6/8 (75.0%)	7/7 (100.0%)	1/2 (50.0%)	0.300
*IKZF1*	1/2 (50.0%)	0/0	1/1 (100.0%)	1.000

^#^Statistically significant difference between the two groups.

Bold values indicates statistically significant *p* values less than 0.05.

Furthermore, the MRD-negative CR rates were 65.3% (96/147) in patients who received CD19 CAR T-cell therapy, 86.3% (44/51) in patients who received tandem CD19/CD22 CAR T-cell therapy, and 76.2% (16/21) in patients who received sequential CD19/CD22 CAR T-cell therapy (*P* = 0.015) (Fig. 2B). Patients over the age of 35 years (56.7% vs. 100.0%, *P* = 0.001), patients without a history of allo-HSCT (65.5% vs. 85.7%, *P* = 0.014), patients with EMD (22.2% vs. 83.3%, *P* = 0.041), patients without a complex karyotype (65.2% vs. 86.7%, *P* = 0.006), and patients with *BCR/ABL1* (60.4% vs. 100.0%, *P* = 0.002) had a greater chance of achieving MRD-negative CR with tandem CD19/CD22 CAR T-cell therapy than with single CD19 CAR T-cell therapy (Supplementary Table 2). Moreover, high-risk patients in the tandem CD19/CD22 group showed a significantly higher MRD-negative CR rate than those in the single CD19 group (92.9% vs. 64.9%, *P* = 0.005) (Fig. 2D). In patients without high-risk factors, there was no significant difference in the MRD-negative CR rate among the three groups (65.8%, 78.3% and 80.0%, respectively; *P* = 0.408) (Fig. 2F).

The univariate analyses revealed that lower frequencies of relapse before CAR T-cell therapy, a lack of evidence indicating EMD and tandem CD19/CD22 CAR T-cell therapy were significantly associated with a superior therapeutic response (Supplementary Table 3–4). In multivariate logistic regression analyses of CR and MRD-negative CR, tandem CD19/CD22 CAR T-cell therapy was also an independent prognostic factor (OR: 0.037, 95% CI: 0.003–0.485, *P* = 0.012; OR: 0.274, 95% CI: 0.108–0.691, *P* = 0.006; respectively) (Table 2). Moreover, EMD and tumor burden before CAR T-cell treatment remained significant independent predictive factors of the CR rate.

**Table 2:** Multivariate analysis for treatment response.

Characteristic	CR		MRD-negative CR	
	*P* value	Exp(B) (95%CI)	*P* value	Exp(B) (95%CI)
Disease status	0.285		0.222	
Refractory*				
First relapse	0.629	1.527 (0.274–8.498)	0.614	0.808 (0.352–1.853)
Second or more relapse	0.211	3.270 (0.511–20.918)	0.366	1.607 (0.574–4.502)
Course of prior therapy				
≥4 vs. ≤3	0.942	1.042 (0.341–3.183)	0.641	0.843 (0.411–1.728)
Prior allo-HSCT				
Yes vs. No	0.471	1.522 (0.486–4.768)	0.747	0.873 (0.384–1.987)
EMD	**<0.001**		**0.029**	
CNSL	**0.002**	35.769 (3.567–358.647)	0.225	3.208 (0.488–21.100)
Other EMD	**<0.001**	63.881 (8.697–469.207)	**0.014**	4.464 (1.351–14.746)
Negative*				
BM blasts by morphology	**0.028**		0.986	
<5%*				
5-20%	**0.014**	9.666 (1.597–58.491)	0.942	0.968 (0.404–2.320)
≥20%	**0.011**	8.360 (1.622–43.096)	0.913	1.041 (0.503–2.155)
High-risk factors				
Yes vs. No	0.569	1.342 (0.488–3.690)	0.712	0.888 (0.471–1.672)
Target	**0.023**		**0.021**	
Single CD19*				
Tandem CD19/CD22	**0.012**	0.037 (0.003–0.485)	**0.006**	0.274 (0.108–0.691)
Sequential CD19/CD22	0.220	0.257 (0.029–2.252)	0.478	0.674 (0.226–2.008)

^*^Control group.

Bold values indicates statistically significant *p* values less than 0.05.

### Tandem CD19/CD22 CAR T-cell therapy showed a similar incidence of adverse events to single CD19 and sequential CD19/CD22 CAR T-cell therapy

All adverse events that occurred during the course of CAR T-cell treatment were graded and are shown in Table 3. In our study, CRS occurred in 168 of 219 patients (76.7%), consisting of 54.3% grade 1–2 and 22.4% severe CRS (grade 3-4). Severe CRS occurred in 25.9% of patients in the single CD19 group, 13.7% of patients in the tandem CD19/CD22 group, and 19.0% in the sequential CD19/CD22 group (*P* = 0.196). A total of 15 patients with ICANS were observed, including 14 patients (9.5%) in the single CD19 group, and 1 patient (2.0%) in the tandem CD19/CD22 group (*P* = 0.108). Three patients harbored HLH, 1 in the single CD19 group, and 2 in the tandem CD19/CD22 group. TLS occurred in 3 patients.

**Table 3:** Adverse events associated to CAR T-cell therapy.

Adverse Event	Single CD19	Tandem CD19/CD22	Sequential CD19/CD22	*P* value
CRS				
Grade 0	37/147 (25.2%)	9/51 (17.6%)	5/21 (23.8%)	0.573
Grade 1-2	72/147 (49.0%)	35/51 (68.6%)	12/21 (57.1%)	0.051
Grade 3-4	38/147 (25.9%)	7/51 (13.7%)	4/21 (19.0%)	0.196
ICANS				
Grade 0	133/147 (90.5%)	50/51 (98.0%)	21/21 (100.0%)	0.108
Grade 1-2	8/147 (5.4%)	1/51 (2.0%)	0/21 (0.0%)	0.470
Grade 3-4	6/147 (4.1%)	0/51 (0.0%)	0/21 (0.0%)	0.406
HLH	1/147 (0.7%)	2/51 (3.9%)	0/21 (0.0%)	0.252
TLS	2/147 (1.4%)	1/51 (2.0%)	0/21 (0.0%)	1.000

Two hundred patients had their levels of serum cytokines monitored regularly from the day of CAR T-cell infusion. Patients in the sequential CD19/CD22 group showed a higher peak of interleukin (IL)-17A than the single CD19 group (*P* = 0.0394 and 0.0054, respectively) (Supplementary Figure 1). There was no significant difference in cytokine levels between the tandem CD19/CD22 group and the single CD19 group.

Regarding hematological toxicity, there was a significant difference in the incidence of severe thrombocytopenia (grade 3-4) among the single CD19, tandem CD19/CD22, and sequential CD19/CD22 groups (44.9%, 58.8% and 71.4%, respectively; *P* = 0.031) but no significant difference in the pairwise comparisons (Supplementary Table 5). Toxicity to other organs was similar among the three groups.

### Allo-HSCT significantly improved the clinical outcomes of patients

**Fig. 3:**
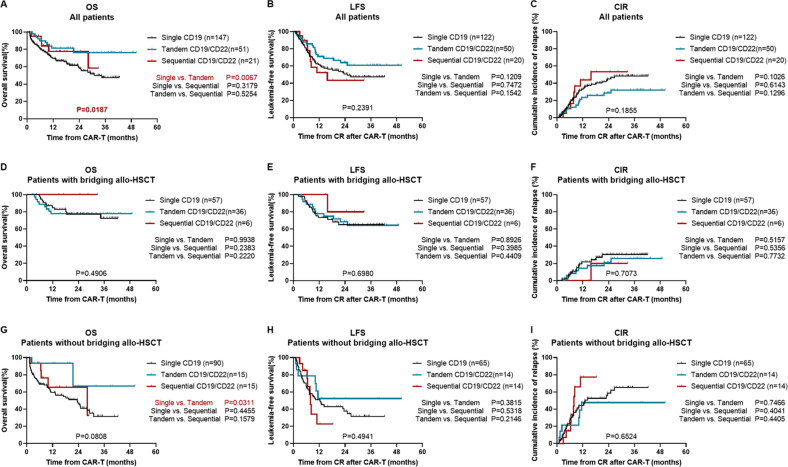
Survival of patients according to different CAR T-cell therapies. **A** Overall survival **B** leukemia-free survival and **C** cumulative incidence of relapse of patients in the single CD19 group, tandem CD19/CD22 group and sequential CD19/CD22 group. **D** Overall survival **E** leukemia-free survival and **F** cumulative incidence of relapse of patients who underwent allo-HSCT after CAR T-cell therapy among the three groups. **G** Overall survival **H** leukemia-free survival and **I** cumulative incidence of relapse of patients without allo-HSCT after CAR T-cell therapy among the three groups.

The median follow-up time across all patients was 25.1 months (range 1.2–50.3 months). The 2-year OS values in the single CD19, tandem CD19/CD22, and sequential CD19/CD22 groups were 59.2%, 76.3%, and 77.6%, respectively (*P* = 0.0187) (Fig. 3A). There was no significant difference in LFS or CIR among the three groups (1-year LFS: 61.7%, 71.1% and 52.2%, respectively, *P* = 0.2391; 1-year CIR: 33.0%, 23.3% and 43.8%, respectively, *P* = 0.1855) (Fig. 3B-C).

Ninety-nine patients who achieved remission with CAR T-cell therapy bridged to allo-HSCT. The allo-HSCT patients showed no significant difference in survival among the single CD19, tandem CD19/CD22, and sequential CD19/CD22 groups (2-year OS: 77.4%, 77.7% and 100.0%, respectively, *P* = 0.4906; 2-year LFS: 65.1%, 68.5% and 80.0%, respectively, *P* = 0.6980; 2-year CIR: 30.5%, 21.2% and 20.0%, respectively, *P* = 0.7073) (Fig. 3D–F). The three groups all had a similar prognosis for post-CAR T-cell therapy bridging to transplantation in patients with or without high-risk factors (Supplementary Fig. 2A–C, Supplementary Fig. 3A–C).

We initially performed univariable analyses to identify baseline and therapy-related factors that were associated with improved LFS in patients who achieved CR and could be included in subsequent multivariable analyses (Supplementary Table 6). Patients who underwent CAR T-cell treatment in refractory and first relapse showed a significantly better LFS than those in second or more relapses (2-year LFS: 61.4%, 57.9% and 32.1%, respectively; *P* = 0.0021) (Supplementary Fig. 4A). The 156 patients who achieved MRD-negative CR showed significantly better median LFS than the 36 patients who achieved MRD -positive CR after CAR T-cell treatment (not reached vs. 9.2 months, *P* = 0.0046) (Supplementary Fig. 4C). Patients who underwent allo-HSCT had better median LFS than patients without allo-HSCT (not reached vs. 10.9 months, *P* < 0.0001) (Supplementary Fig. 4D).

We also performed Cox regression multivariable modeling with the identified variables that had *P* < 0.05 from univariable analyses and clinical factors that could impact survival, such as the number of prior therapies, tumor burden, and high-risk cytogenetics and genetic characteristics. The multivariate Cox model showed that MRD-negative CR (HR: 0.411, 95% CI: 0.240–0.704; *P* = 0.001) and bridging to HSCT (HR: 0.375, 95% CI: 0.234–0.600; *P* < 0.001) were independently associated with better LFS (Table 4). Second or more relapses (HR: 2.644, 95% CI: 1.199–5.832; *P* = 0.016) and a high tumor burden (HR: 1.785, 95% CI: 1.051–3.030; *P* = 0.032) were independent risk factors for LFS.

**Table 4:** Multivariate analysis for LFS.

Characteristic	*P* value	Exp(B)(95% CI)
Disease status	**0.036**	
Refractory*		
First relapse	0.275	1.430 (0.752–2.719)
Second or more relapse	**0.016**	2.644 (1.199–5.832)
Course of prior therapy		
≥4 vs. ≤3	0.753	0.917 (0.533-1.575)
BM blasts by morphology	0.091	
<5%*		
5–20%	0.177	1.555 (0.819–2.951)
≥20%	**0.032**	1.785 (1.051–3.030)
High-risk factors		
Yes vs. No	0.668	1.104 (0.703–1.732)
Response		
MRD-negative CR vs. MRD-positive CR	**0.001**	0.411 (0.240–0.704)
Bridging to HSCT		
Yes vs. No	**<0.001**	0.375 (0.234–0.600)

^*^Control group

### Tandem CD19/CD22 CAR T-cell therapy improved survival for patients without bridging to allo-HSCT

Among patients who did not receive allo-HSCT, 15 patients in the tandem CD19/CD22 group had a significantly longer median OS than the 90 patients in the single CD19 group (not reached vs. 23.5 months, *P* = 0.0311) (Fig. 3G). The median LFS was 12.4 months in the single CD19 group, not reached in the tandem CD19/CD22 group and 7.9 months in the sequential CD19/CD22 group (*P* = 0.4941) (Fig. 3H). Patients with high-risk factors in all three groups had a similar prognosis after CAR T-cell therapy without bridging to transplantation (Supplementary Fig. 2D–F). Patients without high-risk factors in the tandem CD19/CD22 group showed a significantly better OS than those in the single CD19 group (3-year OS: 75.0% vs. 21.2%, *P* = 0.0257) (Supplementary Fig. 3D). There was no difference in survival between the single CD19 and sequential CD19/CD22 CAR T-cell treatments. (Supplementary Fig. 3D–F).

## Discussion

In this study, we compared the efficacy and safety of CD19 single-target and CD19/CD22 dual-target CAR T-cell therapies in a cohort of R/R B-ALL patients. We found that tandem CD19/CD22 CAR T-cell therapy produced a better therapeutic response than single CD19 CAR T-cell therapy, especially in patients with chemotherapy resistance. There was no evidence of increased toxicity associated with the tandem CD19/CD22 CAR T-cell dose. Bridging to transplantation after remission with CAR T-cell therapy significantly improved patient outcomes. Furthermore, tandem CD19/CD22 CAR T-cell therapy was associated with favorable survival for patients without allo-HSCT.

CD19 CAR T-cell therapy has a dramatic effect on R/R B-ALL patients achieving high CR rates, but approximately 20-50% of patients relapse at 6 months [8–11, 27]. To overcome the drawback of single-target CAR T-cell treatment, CD19 and CD22 cells have been applied in combination. Several studies have reported the efficacy of simultaneously targeting multiple antigens in preclinical models using a variety of CAR configurations to achieve recognition of multiple specific antigens [28–33]. Several small samples of clinical data showed that CAR T-cells targeting CD19 and CD22 were clinically active in B-ALL patients [13, 21].

Our data demonstrated that CR was achieved in 98.0% of patients 28 days after tandem CD19/CD22 CAR T-cell infusion. Patients in each high-risk subgroup (complex karyotype, *BCR/ABL1*, Ph-like and *TP53* mutation) benefited from tandem CD19/CD22 CAR T-cell treatment achieving a 100% CR rate. Most importantly, 26 of 28 patients (92.9%) with poor-risk cytogenetic factors achieved MRD-negative CR. Compared with the single CD19 group, the tandem CD19/CD22 group showed an increased MRD-negative CR rate in patients over the age of 35 years, patients without a history of allo-HSCT and patients with EMD. Multivariate analysis confirmed that tandem CD19/CD22 dual-target CAR T-cell therapy was associated with a good treatment response in R/R B-ALL.

Consistent with previous findings, patients with a low tumor burden are more likely to achieve CR compared to patients with a high tumor burden [11, 34, 35]. Therefore, reducing tumor burden including with the use of chemotherapy or targeted therapy prior to CAR T-cell treatment or treating patients in their earlier disease course is important to consider. Furthermore, tandem CD19/CD22 CAR T-cell therapy may offer an additional therapeutic option for patients with chemotherapy resistance. In our study, 25 patients (100.0%) with a high tumor burden achieved CR in the tandem CD19/CD22 group.

Toxicities associated with CAR T-cells such as CRS remain a concern. In most clinical trials, the reported incidence of severe CRS was above 15%, with 13–63% of the patients experiencing neurotoxicity [36–39]. It has been shown that severe CRS is closely related to a high CAR T-cell dose [40]. In our cohort, patients in the tandem CD19/CD22 group received a higher dose of CAR T-cells than patients in the single CD19 group but showed a similar incidence of toxicities.

Despite the initial high CR from CAR T-cell treatment of R/R B-ALL, relapse is still a major problem [10, 11, 41]. No obvious leukemia-free survival advantage was found in patients who received tandem CD19/CD22 CAR T-cell therapy compared with those who received single CD19 or sequential CD19/CD22 CAR T-cell therapy. Multivariable analysis in patients who achieved CR showed that a low frequency of relapse before CAR T-cell therapy, low tumor burden, MRD-negative CR and bridging to HSCT were independently associated with better LFS.

Once patients relapse after allo-HSCT, currently available treatments are often unsatisfactory [42]. However, CAR T-cell therapy is now recognized as holding some promise [8, 9]. Zhang et al. reported that 34 of 43 patients (79.1%) with a history of transplantation achieved CR after CD19 CAR T-cell therapy, and 1-year EFS was 43% [43]. In our patients who relapsed after transplantation, the CR rates were 77.4% (24/31), 88.9% (8/9), and 100.0% (6/6) in the single CD19, tandem CD19/CD22, and sequential CD19/CD22 groups (*P* = 0.605). Among 38 patients who achieved CR, the LFS rate was 59.1% at 2-year. There was no significant difference in survival among the three groups. Overall, patients with a history of transplantation may benefit more from dual-target CAR T-cell therapy.

There is controversy about whether transplantation is necessary after CAR T-cell therapy. Multiple studies have reported that bridging to allo-HSCT after CAR T-cell therapy can improve LFS [35, 44]. However, a recent study suggested no survival benefit from allo-HSCT [11]. In their study, of the 17 patients who underwent transplantation, 6 subsequently relapsed, and another 6 died of transplant-related mortality. When allo-HSCT was incorporated into our LFS multivariable model as a time-dependent covariate, we observed better LFS in CR patients who proceeded to transplantation than in those who did not undergo transplantation.

In conclusion, tandem CD19/CD22 dual-target CAR T-cell therapy obtains a better response than single CD19 CAR T-cell therapy and a similar response to sequential CD19/CD22 CAR T-cell therapy. This provides an effective treatment option for R/R B-ALL patients with high-risk factors. However, our study is limited by the variability in tumor reduction chemotherapy regimens, the small sample size and short follow-up duration of the sequential CD19/CD22 CAR T-cell group, and the difference in CAR T-cell infusion doses among the three groups. Data from large prospective, randomized, and controlled clinical trials are required to verify the superiority of dual-target CAR T-cell therapy. Additionally, efforts should be made to improve the response duration.

## Supplementary information

Supplementary Information

## Data Availability

The data that support the findings of this study are available from the corresponding author upon reasonable request.
