# Supplementary material for: Which one is better for refractory/relapsed acute B-cell lymphoblastic leukemia: Single-target (CD19) or dual-target (tandem or sequential CD19/CD22) CAR T-cell therapy?
Source: Blood Cancer J. 2023 Apr 24;13(1):60. doi: 10.1038/s41408-023-00819-5 (PMC10125987; doi:10.1038/s41408-023-00819-5)

**Supplementary Table 1. Baseline characteristics of patients**

| **Characteristic** | **All patients**  **n=219** | **Single**  **CD19**  **n=147** | **Tandem CD19/CD22**  **n=51** | **Sequential CD19/CD22**  **n=21** | **P value** |
| --- | --- | --- | --- | --- | --- |
|  | N (%) | n/N (%) | n/N (%) | n/N (%) |  |
| **Gender** |  |  |  |  | 0.184 |
| Male | 106(48.4%) | 68(46.3%) | 30(58.8%) | 8(38.1%) |  |
| Female | 113(51.6%) | 79(53.7%) | 21(41.2%) | 13(61.9%) |  |
| **Age (y)** |  |  |  |  | 0.456 |
| ≤14 | 20(9.1%) | 11(7.5%) | 7(13.7%) | 2(9.5%) |  |
| 15-34 | 107(48.9%) | 69(46.9%) | 26(51.0%) | 12(57.1%) |  |
| ≥35 | 92(42.0%) | 67(45.6%) | 18(35.3%) | 7(33.3%) |  |
| **Disease status** |  |  |  |  | 0.525 |
| Refractory | 50(22.8%) | 32(21.8%) | 15(29.4%) | 3(14.3%) |  |
| First relapse | 120(54.8%) | 80(54.4%) | 28(54.9%) | 12(57.1%) |  |
| Second or more relapse | 49(22.4%) | 35(23.8%) | 8(15.7%) | 6(28.6%) |  |
| **Course of prior therapy** |  |  |  |  | 0.211 |
| ≤3 | 101(46.1%) | 72(49.0%) | 23(45.1%) | 6(28.6%) |  |
| ≥4 | 118(53.9%) | 75(51.0%) | 28(54.9%) | 15(71.4%) |  |
| **Prior allo-HSCT** |  |  |  |  | 0.570 |
| Yes | 46(21.0%) | 31(21.1%) | 9(17.6%) | 6(28.6%) |  |
| No | 173(79.0%) | 116(78.9%) | 42(82.4%) | 15(71.4%) |  |
| **EMD** |  |  |  |  | 0.285 |
| CNSL | 5(2.3%) | 5(3.4%) | 0(0.0%) | 0(0.0%) |  |
| Other EMD | 15(6.8%) | 9(6.1%) | 6(11.8%) | 0(0.0%) |  |
| Negative | 199(90.9%) | 133(90.5%) | 45(88.2%) | 21(100.0%) |  |
| **BM blasts by morphology** |  |  |  |  | 0.562 |
| <5% | 90(41.1%) | 64(43.5%) | 19(37.3%) | 7(33.3%) |  |
| 5-20% | 40(18.3%) | 28(19.0%) | 7(13.7%) | 5(23.8%) |  |
| ≥20% | 89(40.6%) | 55(37.4%) | 25(49.0%) | 9(42.9%) |  |
| **Complex karyotype** |  |  |  |  | 0.223 |
| Yes | 22(10.0%) | 12(8.2%) | 6(11.8%) | 4(19.0%) |  |
| No | 197(90.0%) | 135(91.8%) | 45(88.2%) | 17(81.0%) |  |
| **Fusion gene** |  |  |  |  |  |
| *BCR-ABL1* | 64(29.2%) | 48(32.7%) | 16(31.4%) | 0(0.0%) | **0.008** |
| Ph-like | 10(4.6%) | 4(2.7%) | 5(9.8%) | 1(4.8%) | 0.091 |
| *KMT2A* rearrangement | 12(5.5%) | 9(6.1%) | 1(2.0%) | 2(9.5%) | 0.347 |
| Other fusion genes | 12(5.5%) | 6(4.1%) | 4(7.8%) | 2(9.5%) | 0.308 |
| Negative | 121(55.3%) | 80(54.4%) | 25(49.0%) | 16(76.2%) | 0.102 |
| **Gene mutation** |  |  |  |  |  |
| *T315I* | 30(13.7%) | 25(17.0%) | 5(9.8%) | 0(0.0%) | 0.061 |
| *TP53* | 17(7.8%) | 8(5.4%) | 7(13.7%) | 2(9.5%) | 0.125 |
| *IKZF1* | 3(1.4%) | 2(1.4%) | 0(0.0%) | 1(4.8%) | 0.383 |

**Supplementary Table 2. Subgroup analysis of MRD-negative CR rate**

| **Characteristic** | **Single**  **CD19**  **n=147** | **Tandem CD19/CD22**  **n=51** | **Sequential CD19/CD22**  **n=21** | **P value** |
| --- | --- | --- | --- | --- |
|  | n/N (%) | n/N (%) | n/N (%) |  |
| **Gender** |  |  |  |  |
| Male | 44/68(64.7%) | 26/30(86.7%) | 5/8(62.5%) | 0.054 |
| Female | 52/79(65.8%) | 18/21(85.7%) | 11/13(84.6%) | 0.137 |
| **Age (y)** |  |  |  |  |
| ≤14 | 9/11(81.8%) | 6/7(85.7%) | 2/2(100.0%) | 1.000 |
| 15-34 | 49/69(71.0%) | 20/26(76.9%) | 9/12(75.0%) | 0.897 |
| ≥35 | 38/67(56.7%)^#^ | 18/18(100.0%)^#^ | 5/7(71.4%) | **<0.001** |
| **Disease status** |  |  |  |  |
| Refractory | 20/32(62.5%) | 13/15(86.7%) | 3/3(100.0%) | 0.164 |
| First relapse | 55/80(68.8%)^#^ | 26/28(92.9%)^#^ | 9/12(75.0%) | **0.029** |
| Second or more relapse | 21/35(60.0%) | 5/8(62.5%) | 4/6(66.7%) | 1.000 |
| **Course of prior therapy** |  |  |  |  |
| ≤3 | 46/72(63.9%) | 20/23(87.0%) | 5/6(83.3%) | 0.075 |
| ≥4 | 50/75(66.7%) | 24/28(85.7%) | 11/15(73.3%) | 0.168 |
| **Prior allo-HSCT** |  |  |  |  |
| Yes | 20/31(64.5%) | 8/9(88.9%) | 5/6(83.3%) | 0.379 |
| No | 76/116(65.5%)^#^ | 36/42(85.7%)^#^ | 11/15(73.3%) | **0.041** |
| **EMD** |  |  |  |  |
| CNSL | 2/5(40.0%) | 0/0 | 0/0 | — |
| Other EMD | 2/9(22.2%)^#^ | 5/6(83.3%)^#^ | 0/0 | **0.041** |
| Negative | 92/133(69.2%) | 39/45(86.7%) | 16/21(76.2%) | 0.067 |
| **BM blasts by morphology** |  |  |  |  |
| <5% | 41/64(64.1%) | 17/19(89.5%) | 4/7(57.1%) | 0.062 |
| 5-20% | 19/28(67.9%) | 5/7(71.4%) | 5/5(100.0%) | 0.463 |
| ≥20% | 36/55(65.5%) | 22/25(88.0%) | 7/9(77.8%) | 0.092 |
| **Complex karyotype** |  |  |  |  |
| Yes | 8/12(66.7%) | 5/6(83.3%) | 2/4(50.0%) | 0.598 |
| No | 88/135(65.2%)^#^ | 39/45(86.7%)^#^ | 14/17(82.4%) | **0.012** |
| **Fusion gene** |  |  |  |  |
| *BCR-ABL1* | 29/48(60.4%)^#^ | 16/16(100.0%)^#^ | 0/0 | **0.002** |
| Ph-like | 4/4(100.0%) | 3/5(60.0%) | 1/1(100.0%) | 0.556 |
| *KMT2A* rearrangement | 6/9(66.7%) | 1/1(100.0%) | 2/2(100.0%) | 1.000 |
| Other fusion genes | 4/6(66.7%) | 4/4(100.0%) | 1/2(50.0%) | 0.345 |
| Negative | 53/80(66.3%) | 20/25(80.0%) | 12/16(75.0%) | 0.401 |
| **Gene mutation** |  |  |  |  |
| *T315I* | 14/25(56.0%) | 5/5(100.0%) | 0/0 | 0.129 |
| *TP53* | 5/8(62.5%) | 7/7(100.0%) | 1/2(50.0%) | 0.165 |
| *IKZF1* | 1/2(50.0%) | 0/0 | 0/1(0.0%) | 1.000 |

# Statistically significant difference between the two groups.

**Supplementary Table 3. Univariate analysis for CR rate**

| **Characteristic** | **CR rate** | **P value** | **Exp(B)(95%CI)** |
| --- | --- | --- | --- |
| **Gender** |  |  |  |
| Male* | 90/106(84.9%) |  |  |
| Female | 102/113(90.3%) | 0.231 | 0.607(0.268-1.375) |
| **Age (y)** |  | 0.605 |  |
| ≤14* | 19/20(95.0%) |  |  |
| 15-34 | 93/107(86.9%) | 0.324 | 2.860(0.355-23.076) |
| ≥35 | 80/92(87.0%) | 0.328 | 2.850(0.349-23.283) |
| **Disease status** |  | **0.031** |  |
| Refractory* | 48/50(96.0%) |  |  |
| First relapse | 106/120(88.3%) | 0.137 | 3.170(0.693-14.498) |
| Second or more relapse | 38/49(77.6%) | **0.015** | 6.947(1.452-33.247) |
| **Course of prior therapy** |  |  |  |
| ≤3* | 91/101(90.1%) |  |  |
| ≥4 | 101/118(85.6%) | 0.315 | 1.532(0.667-3.516) |
| **Prior allo-HSCT** |  |  |  |
| Yes | 38/46(82.6%) | 0.244 | 1.706(0.694-4.193) |
| No* | 154/173(89.0%) |  |  |
| **EMD** |  | **<0.001** |  |
| CNSL | 2/5(40.0%) | **0.003** | 16.059(2.508-102.844) |
| Other EMD | 8/15(53.3%) | **<0.001** | 9.368(3.027-28.988) |
| Negative* | 182/199(91.5%) |  |  |
| **BM blasts by morphology** |  | 0.439 |  |
| <5%* | 82/90(91.1%) |  |  |
| 5-20% | 34/40(85.0%) | 0.305 | 1.809(0.583-5.508) |
| ≥20% | 76/89(85.4%) | 0.239 | 1.753(0.689-4.463) |
| **Complex karyotype** |  |  |  |
| Yes | 19/22(86.4%) | 0.844 | 1.138(0.313-4.136) |
| No* | 173/197(87.8%) |  |  |
| **Fusion gene** |  | 0.983 |  |
| *BCR-ABL1* | 56/64(87.5%) | 0.889 | 0.938(0.378-2.326) |
| Ph-like | 10/10(100.0%) | 0.999 | — |
| *KMT2A* rearranged | 11/12(91.7%) | 0.632 | 0.597（0.072-4.939） |
| Other fusion genes | 10/12(83.3%) | 0.740 | 1.313（0.263-6.545） |
| Negative* | 105/121(86.8%) |  |  |
| **Gene mutation** |  |  |  |
| *T315I* | 26/30(86.7%) | 0.857 | 1.110(0.355-3.470) |
| *TP53* | 14/17(82.4%) | 0.491 | 1.589(0.426-5.836) |
| *IKZF1* | 2/3(66.7%) | 0.297 | 3.654(0.320-41.720) |
| **Target** |  | **0.036** |  |
| Single CD19* | 122/147(83.0%) |  |  |
| Tandem CD19/CD22 | 50/51(98.0%) | **0.024** | 0.098(0.013-0.740) |
| Sequential CD19/CD22 | 20/21(95.2%) | 0.178 | 0.244(0.031-1.903) |

* Control group

**Supplementary Table 4. Univariate analysis for MRD-negative CR rate**

| **Characteristic** | **CR rate** | **P value** | **Exp(B)(95%CI)** |
| --- | --- | --- | --- |
| **Gender** |  |  |  |
| Male* | 75/106(70.8%) |  |  |
| Female | 81/113(71.7%) | 0.883 | 1.045(0.582-1.877) |
| **Age (y)** |  | 0.228 |  |
| ≤14* | 17/20(85.0%) |  |  |
| 15-34 | 78/107(72.9%) | 0.261 | 2.107(0.575-7.725) |
| ≥35 | 61/92(66.3%) | 0.111 | 2.880(0.784-10.580) |
| **Disease status** |  | 0.203 |  |
| Refractory* | 36/50(70.0%) |  |  |
| First relapse | 90/120(75.0%) | 0.684 | 0.857(0.408-1.802) |
| Second or more relapse | 30/49(63.3%) | 0.257 | 1.629(0.701-3.785) |
| **Course of prior therapy** |  |  |  |
| ≤3* | 71/101(70.3%) |  |  |
| ≥4 | 85/118(72.0%) | 0.777 | 0.919(0.511-1.651) |
| **Prior allo-HSCT** |  |  |  |
| Yes | 33/46(71.7%) | 0.932 | 0.969(0.471-1.993) |
| No* | 123/173(71.1%) |  |  |
| **EMD** |  | **0.034** |  |
| CNSL | 2/5(40.0%) | 0.119 | 4.240(0.689-26.091) |
| Other EMD | 7/15(46.7%) | **0.031** | 3.231(1.116-9.349) |
| Negative* | 147/199(73.9%) |  |  |
| **BM blasts by morphology** |  | 0.813 |  |
| <5%* | 62/90(68.9%) |  |  |
| 5-20% | 29/40(72.5%) | 0.679 | 0.840(0.368-1.917) |
| ≥20% | 65/89(73.0%) | 0.542 | 0.818(0.428-1.561) |
| **Complex karyotype** |  |  |  |
| Yes | 15/22(68.2%) | 0.739 | 1.175(0.455-3.035) |
| No* | 141/197(71.6%) |  |  |
| **Fusion gene** |  | 0.961 |  |
| *BCR-ABL1* | 45/64(70.3%) | 0.993 | 0.997(0.514-1.934) |
| Ph-like | 8/10(80.0%) | 0.518 | 0.590(0.119-2.917) |
| *KMT2A* rearranged | 9/12(75.0%) | 0.731 | 0.787(0.201-3.077) |
| Other fusion genes | 9/12(75.0%) | 0.731 | 0.787(0.201-3.077) |
| Negative* | 85/121(70.2%) |  |  |
| **Gene mutation** |  |  |  |
| *T315I* | 19/30(63.3%) | 0.306 | 1.525(0.680-3.423) |
| *TP53* | 13/17(76.5%) | 0.620 | 0.746(0.234-2.381) |
| *IKZF1* | 1/3(33.3%) | 0.188 | 5.082(0.452-57.075) |
| **Target** |  | **0.020** |  |
| Single CD19* | 96/147(65.3%) |  |  |
| Tandem CD19/CD22 | 44/51(86.3%) | **0.006** | 0.299(0.126-0.713) |
| Sequential CD19/CD22 | 16/21(76.2%) | 0.327 | 0.588(0.204-1.698) |

* Control group

**Supplementary Table 5. Organ toxicity associated to CAR T-cell therapy**

| **Characteristic** | **Grade** | **Single**  **CD19** | **Tandem CD19/CD22** | **Sequential CD19/CD22** | **P value** |
| --- | --- | --- | --- | --- | --- |
| **Hematological Toxicity** |  |  |  |  |  |
| Myelosuppression |  |  |  |  |  |
| Neutrophil granulocyte | 1-2 | 20/147(13.6%) | 7/51(13.7%) | 0/21(0.0%) | 0.183 |
|  | 3-4 | 117/147(79.6%) | 38/51(74.5%) | 20/21(95.2%) | 0.130 |
| Hemoglobin | 1-2 | 50/147(34.0%) | 13/51(25.5%) | 4/21(19.0%) | 0.252 |
|  | 3-4 | 92/147(62.6%) | 35/51(68.6%) | 15/21(71.4%) | 0.592 |
| Platelet | 1-2 | 41/147(27.9%) | 13/51(25.5%) | 2/21(9.5%) | 0.196 |
|  | 3-4 | 66/147(44.9%) | 30/51(58.8%) | 15/21(71.4%) | **0.031** |
| Coagulation abnormality | 1-2 | 61/147(41.5%) | 25/51(49.0%) | 10/21(47.6%) | 0.605 |
|  | 3-4 | 1/147(0.7%) | 0/51(0.0%) | 0/21(0.0%) | 1.000 |
| Bleeding | 1-2 | 6/147(4.1%) | 3/51(5.9%) | 0/21(0.0%) | 0.654 |
|  | 3-4 | 1/147(0.7%) | 0/51(0.0%) | 0/21(0.0%) | 1.000 |
| **Non-hematological Toxicity** |  |  |  |  |  |
| Infection | 1-2 | 19/147(12.9%) | 4/51(7.8%) | 3/21(14.3%) | 0.576 |
|  | 3-4 | 23/147(15.6%) | 13/51(25.5%) | 5/21(23.8%) | 0.246 |
| Gastrointestinal reaction | 1-2 | 13/147(8.8%) | 2/51(3.9%) | 2/21(9.5%) | 0.608 |
|  | 3-4 | 2/147(1.4%) | 1/51(2.0%) | 0/21(0.0%) | 1.000 |
| Cardiotoxicity | 3-4 | 7/147(4.8%) | 2/51(3.9%) | 1/21(4.8%) | 1.000 |
| Hepatotoxicity | 1-2 | 40/147(27.2%) | 15/51(29.4%) | 6/21(28.6%) | 0.953 |
|  | 3-4 | 19/147(12.9%) | 4/51(7.8%) | 1/21(4.8%) | 0.513 |
| Renal toxicity | 1-2 | 14/147(9.5%) | 7/51(13.7%) | 2/21(9.5%) | 0.693 |
|  | 3-4 | 1/147(0.7%) | 0/51(0.0%) | 0/21(0.0%) | 1.000 |

**Supplementary Table 6. Univariate analysis for LFS**

| **Characteristic** | **2-year LFS** | **P value** | **Exp(B)(95%CI)** |
| --- | --- | --- | --- |
| **Gender** |  |  |  |
| Male* | 51.5% |  |  |
| Female | 56.2% | 0.344 | 0.810(0.523-1.254) |
| **Age (y)** |  | 0.477 |  |
| ≤14* | 46.3% |  |  |
| 15-34 | 60.5% | 0.289 | 0.694(0.353-1.364) |
| ≥35 | 48.1% | 0.677 | 0.865(0.438-1.708) |
| **Disease status** |  | **0.003** |  |
| Refractory* | 61.4% |  |  |
| First relapse | 57.9% | 0.397 | 1.282(0.722-2.277) |
| Second or more relapse | 32.1% | **0.002** | 2.765(1.443-5.299) |
| **Course of prior therapy** |  |  |  |
| ≤3* | 57.9% |  |  |
| ≥4 | 50.3% | 0.133 | 1.403(0.902-2.182) |
| **Prior allo-HSCT** |  |  |  |
| Yes | 59.1% | 0.789 | 0.926(0.529-1.623) |
| No* | 52.7% |  |  |
| **EMD** |  |  |  |
| Yes | 57.9% | 0.403 | 0.611(0.193-1.937) |
| No* | 53.5% |  |  |
| **BM blasts by morphology** |  | 0.269 |  |
| <5%* | 59.5% |  |  |
| 5-20% | 49.5% | 0.443 | 1.276(0.684-2.383) |
| ≥20% | 50.0% | 0.106 | 1.494(0.919-2.430) |
| **Complex karyotype** |  |  |  |
| Yes | 33.7% | 0.146 | 1.603(0.848-3.032) |
| No* | 56.1% |  |  |
| **Fusion gene** |  | 0.935 |  |
| *BCR-ABL1* | 54.6% | 0.916 | 0.973(0.589-1.609) |
| Ph-like | 45.0% | 0.721 | 1.183(0.470-2.982) |
| *KMT2A* rearranged | 46.8% | 0.511 | 1.364(0.540-3.441) |
| Other fusion genes | 80.0% | 0.662 | 0.771(0.239-2.481) |
| Negative* | 53.1% |  |  |
| **Gene mutation** |  |  |  |
| *T315I* | 47.2% | 0.323 | 1.349(0.745-2.442) |
| *TP53* | 57.1% | 0.678 | 0.838(0.365-1.926) |
| **Target** |  | 0.245 |  |
| Single CD19* | 50.7% |  |  |
| Tandem CD19/CD22 | 63.9% | 0.121 | 0.655(0.384-1.118) |
| Sequential CD19/CD22 | 43.5% | 0.733 | 1.131(0.558-2.293) |
| **Response** |  |  |  |
| MRD-negative CR | 58.4% | **0.005** | 0.499(0.306-0.815) |
| MRD-positive CR* | 35.1% |  |  |
| **Bridging to HSCT** |  |  |  |
| Yes | 67.5% | **<0.001** | 0.356(0.226-0.560) |
| No* | 37.8% |  |  |
| **CRS** |  | 0.406 |  |
| Grade 0* | 58.4% |  |  |
| Grade 1-2 | 55.6% | 0.618 | 1.151(0.663-1.999) |
| Grade 3-4 | 44.3% | 0.197 | 1.514(0.806-2.843) |

* Control group

**Supplementary Figure 1. Peak of cytokine levels.**

The peak concentration of serum Fer, CRP, IL-2, IL-4, IL-6, IL-10, TNF-α and IFN-γ in the single CD19, tandem CD19/CD22 and sequential CD19/CD22 group.


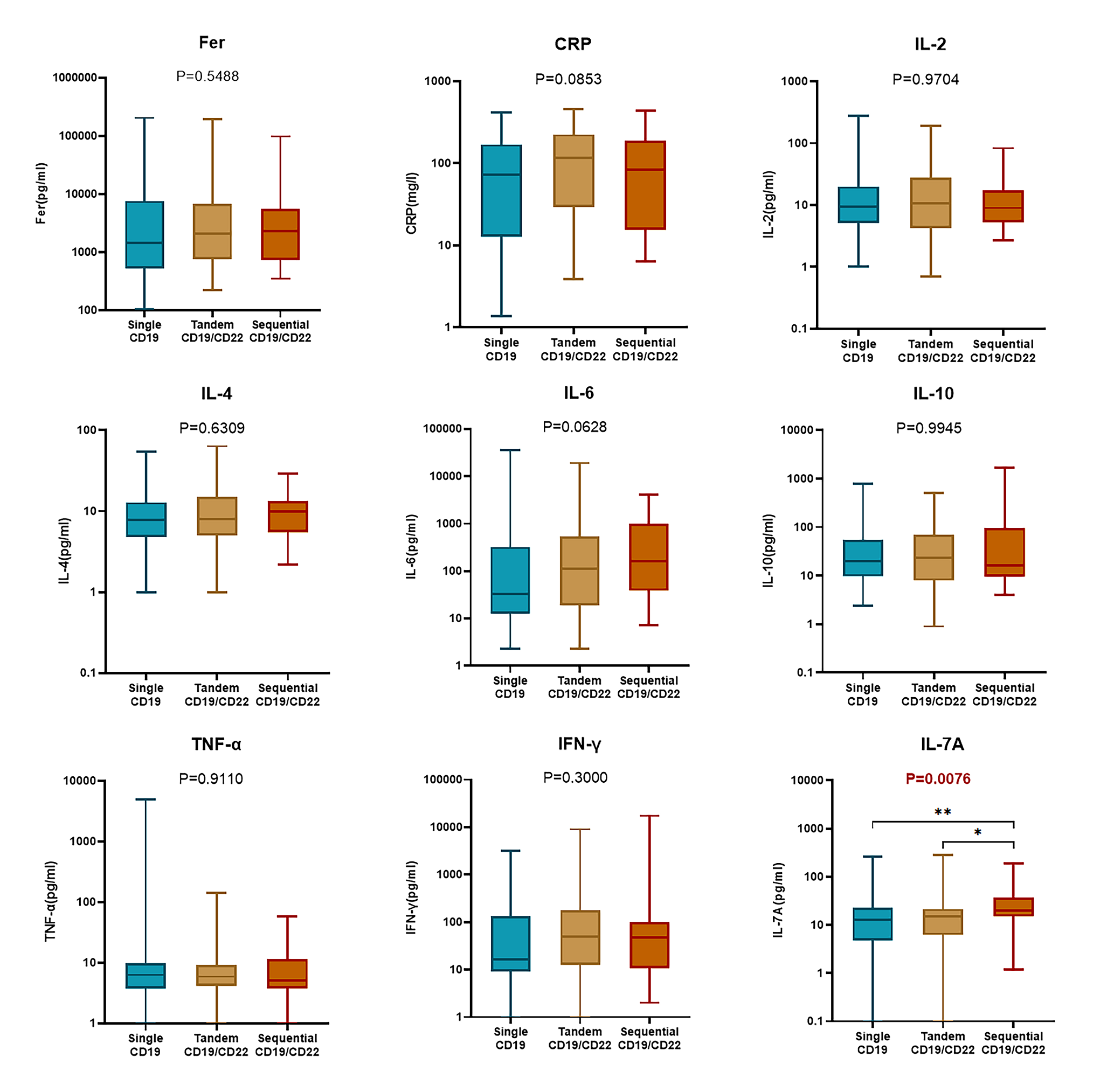


**Supplementary Figure 2. Survival of patients with high-risk factors according to different CAR T-cell therapies.**

(A) Overall survival (B) leukemia-free survival and (C) cumulative incidence of relapse of patients who underwent allo-HSCT after CAR T-cell therapy in the single CD19 group, tandem CD19/CD22 group and sequential CD19/CD22 group.

(D) Overall survival (E) leukemia-free survival and (F) cumulative incidence of relapse of patients without allo-HSCT after CAR T-cell therapy among the three groups.


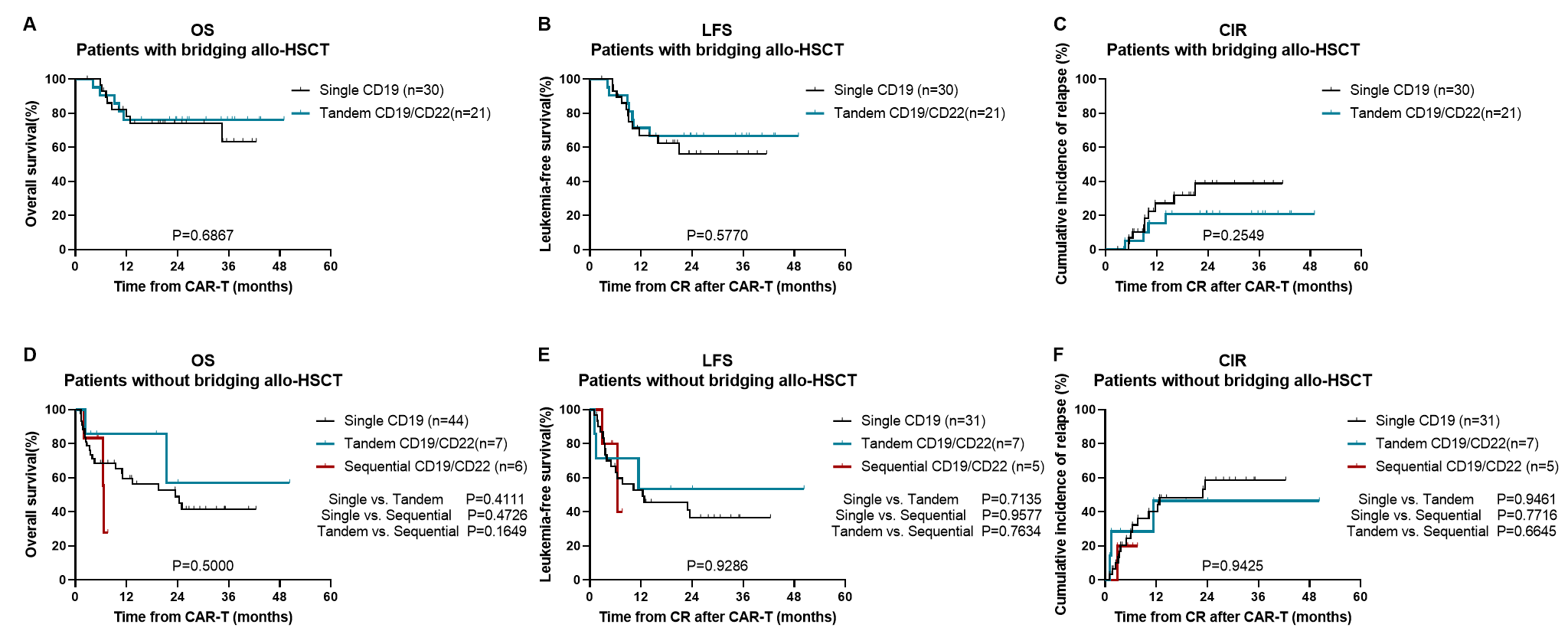


**Supplementary Figure 3. Survival of patients without high-risk factors according to different CAR T-cell therapies.**

(A) Overall survival (B) leukemia-free survival and (C) cumulative incidence of relapse of patients who underwent allo-HSCT after CAR T-cell therapy in the single CD19 group, tandem CD19/CD22 group and sequential CD19/CD22 group.

(D) Overall survival (E) leukemia-free survival and (F) cumulative incidence of relapse of patients without allo-HSCT after CAR T-cell therapy among the three groups.


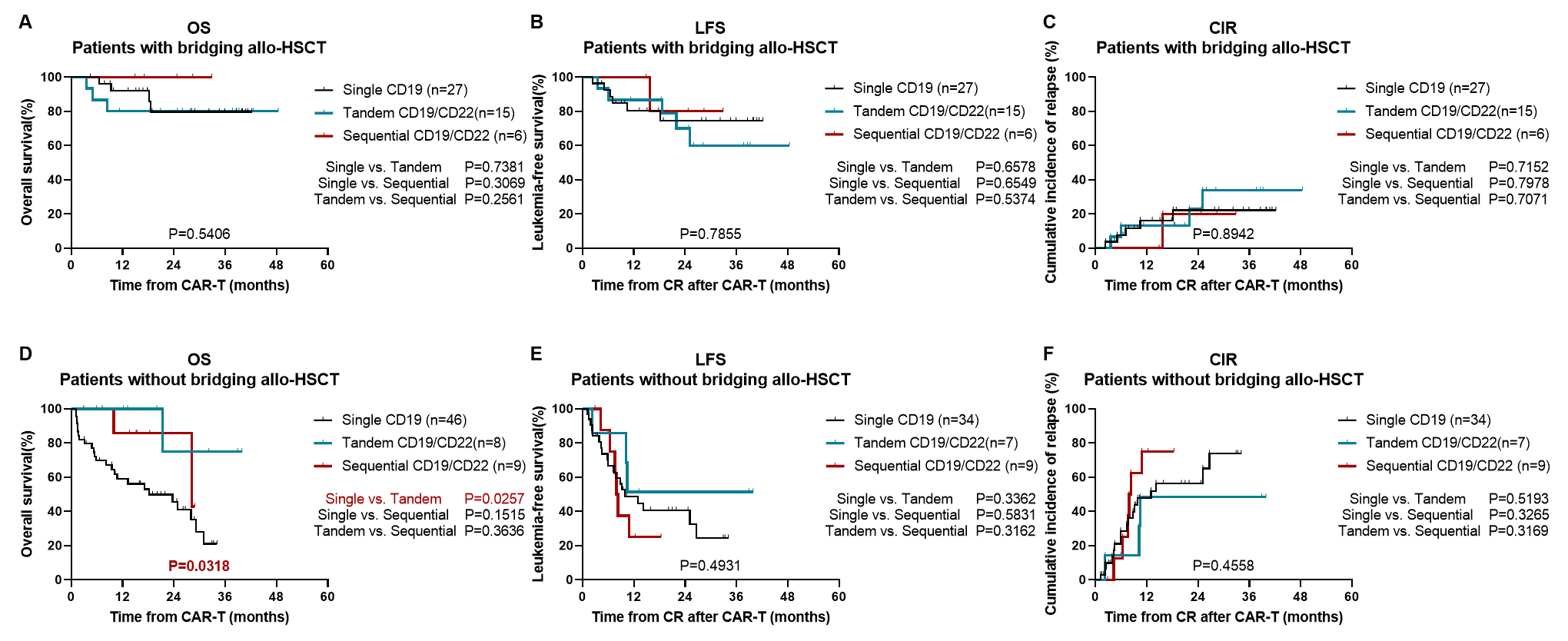


**Supplementary Figure 4. Survival of the entire study population.**

1. Leukemia-free survival of patients according to disease status.
2. Leukemia-free survival of patients according tumor burden.
3. Leukemia-free survival of patients according to response.
4. Leukemia-free survival of patients according to bridging to allo-HSCT.


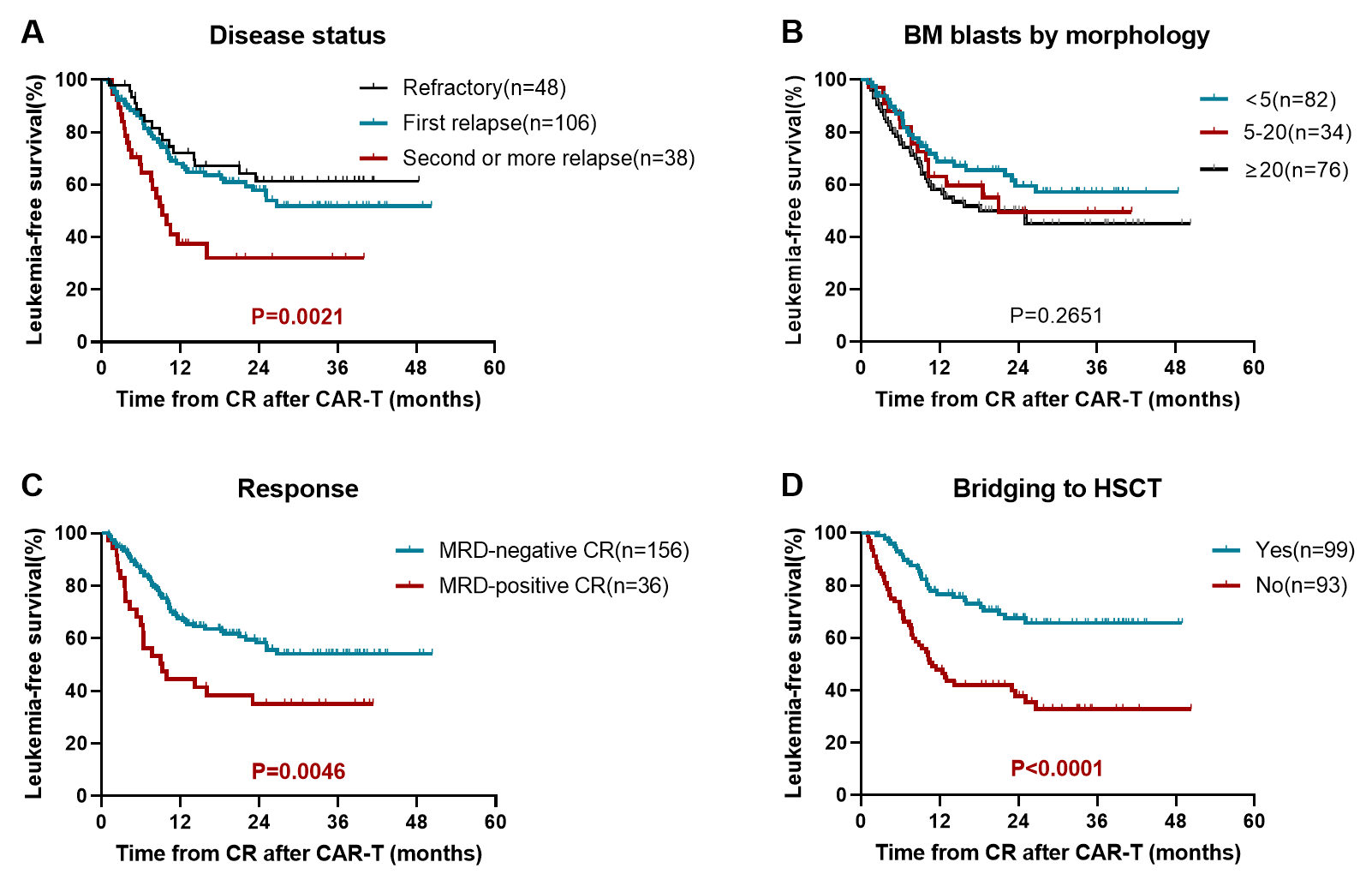

Supplement: Supplementary file 1 — Supplementary Information [file 41408_2023_819_MOESM1_ESM.docx]
